# The desirability of education in didactic skills according to medical interns

**DOI:** 10.1007/s40037-012-0036-x

**Published:** 2012-11-28

**Authors:** Anne T. Kloek, Joshua R. A. Verbakel, Simone E. Bernard, Januska Evenboer, Eef J. Hendriks, Hanneke Stam

**Affiliations:** 1 Willem de Zwijgerstraat 8, 3583 HC Utrecht, the Netherlands; 2 Huijbersstraat 187, 6524 PA Nijmegen, the Netherlands; 3 Rochussenstraat 395D, 3023 DK Rotterdam, the Netherlands; 4 Gansstraat 14a, 3582 EH Utrecht, the Netherlands; 5 Bijleveldsingel 70 K12, 6524 AE Nijmegen, the Netherlands; 6 Noordkaapstraat 9, 1013 CE Amsterdam, the Netherlands

**Keywords:** Education, Medicine, Didactic skills, Students, Medical internships

## Abstract

Since all doctors at some point in their career will be faced with their role as a teacher, it appears desirable that future doctors are educated in didactic skills. At present, however, there are no formal opportunities for developing didactic skills at the majority of Dutch medical faculties. The main question of this study is: How do medical interns perceive the quality and quantity of their education in didactic skills? The Dutch Association for Medical Interns (LOCA) ran a national survey among 1,008 medical interns that measured the interns’ self-assessed needs for training in didactic skills during medical school. Almost 80 % of the respondents argue that the mastery of didactic skills composes an essential competency for doctors, with the skill of providing adequate feedback considered to be the most important didactic quality for doctors. Of the respondents, 41 % wish to be educated in didactic skills, both during their medical undergraduate degree and during their subsequent training to become a resident. Teaching while being observed and receiving feedback in this setting is regarded as a particularly valuable didactic method by 74 % of the medical interns. Of the respondents, 82 % would invest time to follow training for the development of didactic skills if it was offered. Medical interns stress the importance of doctors’ didactic skills during their clinical internships. Compared with current levels, most interns desire increased attention to the formal development of didactic skills during medical school. Considering the importance of didactic skills and the need for more extensive training, the LOCA advises medical faculties to include more formal didactic training in the medical curriculum.

## Introduction

The Hippocratic Oath states: ‘I shall promote the medical knowledge of myself and others’ [1]. Effectively, this anchors didactic skills in the Oath and not without reason. Excellent didactics allow medical students to gain knowledge faster. Moreover, didactic skills come to use when interacting with patients [2]. Despite a growing interest in medical education and an increase in the pursuit of formal training to enhance their ability as teachers, not every doctor has a didactic approach. In addition, the development of didactic skills lacks attention in the Dutch medical curriculum. At present, most of the eight Dutch medical faculties present little to no opportunity for developing didactic skills [3]. In fact, with the exception of Utrecht University, at the time of writing no Dutch medical school spends more than ten obligatory hours on education in didactic skills during the six undergraduate years [3]. Utrecht University offers a formal peer-assisted learning programme. In this compulsory programme sixth-year medical students take a one-week course in teacher training which includes practical teaching experience [4].

Time spent on the development of didactic skills in Dutch medical degrees is also considerably lower compared with other countries. In the United States, 44 % of medical schools offer student-as-teacher programmes to train medical students in their roles as future teachers, most offering this in the senior year [5].

Training programmes in didactic skills prepare students for their future roles as residents or faculty members and have a number of benefits. Medical students develop into better educators after participating in a programme for teacher preparation [6]. In addition, medical students can become more effective communicators when interacting with patients as teaching is an essential aspect of this interaction [7].

Furthermore, when medical interns develop a better understanding of teaching and learning principles they may also become better students [7, 8]. Finally, didactic skills programmes, such as the Utrecht student-as-teacher programme, provide the medical faculty with a valuable supply of teaching assistance.

During medical internships, varying in length between 2 weeks and 3 months, medical students mainly receive training from doctors. A large group of the teachers have often just finished medical school and frequently have not received any formal training in didactic skills.

The current study will evaluate the perceived need for more and better preparation in didactic training among medical interns. The main question of this study is therefore:

How do medical interns perceive the quality and quantity of their education in didactic skills?

The study also aims to provide recommendations where necessary for the improvement of training in didactic skills.

## Methods

A digital survey was distributed among all Dutch medical schools. In most of the schools, interns are fourth- to sixth-year students. Only in Utrecht do medical undergraduate students start their internships in their third year.

The survey was designed by the Education Officer of the LOCA board 2011 using the online survey programme Thesistools.com. Before distributing the survey it was pilot tested in a small pool of students (*n* = 10) to ensure understandable, neutral and specific wording in the survey [9]. The link to the final version of the survey was distributed at a LOCA conference, via the LOCA website, the LOCA newsletter and where possible via student email and online communities of the various medical schools.

The survey defined ‘didactic skills’ as follows: *The ability to pass on knowledge, skills and insight* [10]. The survey consisted of two open and 16 multiple choice questions regarding education in didactic skills with the possibility to add remarks. The survey included questions that could be answered factually, such as ‘is education in didactic skills part of the educational programme of the medical school you attend’ and questions demanding a more subjective response, such as ‘which didactic skills are important?’. The answer scale varied. Statements were either presented with ‘agree’, ‘disagree’, ‘no opinion’, whereas other questions had a scale of six possible answers.

For initial analysis of data, Thesistool.com was used. Further analysis of the data was carried out in SPSS Statistics 17.0.

## Results

### Respondents

A total of 1,008 medical interns participated in the current study. Of these 86.4 % had been doing internships for more than 6 months at the time of the study.

At the time of the study there were approximately 6,500 medical interns in the Netherlands (source: Dutch capacity institution), amounting to a response rate of 15.5 %. Respondents from Utrecht, Groningen and Nijmegen were overrepresented. The response rates for these faculties were supposedly higher because the study could be distributed via student email and/or blackboard. Because of this, the results are not representative of the general population of medical students for all eight medical schools.

The respondents were equally distributed per study year; 24.7 % were students in year 4, 36.7 % in year 5 and 31.9 % in year 6. The remaining of 6.7 % did not fit in one of those study years and were classified as ‘other study year’.

### The importance of didactic skills

**Fig. 1:**
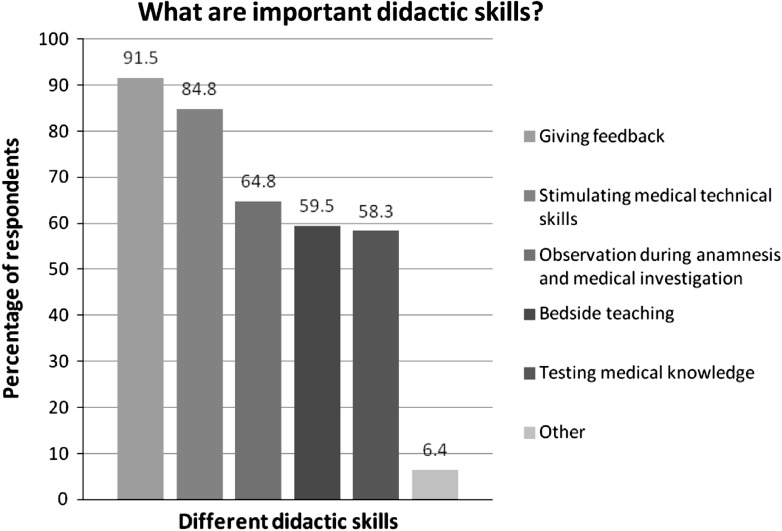
Important didactic skills

Figure 1 shows the didactic skills considered important by medical interns. Giving feedback is thought to be the most important didactic skill by 91.5 % of the respondents. This is followed by stimulating medical technical skills and observation during anamnesis and physical investigation by 84.8 and 64.8 % of respondents, respectively. Respondents regularly mentioned that, in addition to the given options, giving explanations and stimulating clinical reasoning are also important didactic skills.

Of the respondents, 84.9 % agreed that mastering didactic skills is one of the basic skills of a resident. Medical interns consider good guidance important for a sufficiently high return from their internship in terms of learning. Of the questioned medical interns, 85.9 % pointed out the need for didactically minded doctors and they argued that interns can not be held completely responsible for their own learning.

#### Education in didactic skills

##### Is it necessary?

Most respondents, 78.2 %, considered it necessary to master didactic skills in order to be a proficient doctor. In addition, 84.3 % believe that doctors should be given additional training in didactic skills in order for them to be more education minded when dealing with interns.

Opinions on the timing of training varied: 41.0 % thought that training in didactic skills should be provided both during the undergraduate curriculum to medical students as well as during residency training, whereas 38.3 % thought this type of training should only be given while already working as a resident. In contrast, 15.8 % thought only medical interns should be given this kind of training before taking up residency and a mere 3.3 % found no need for this type of training.

##### Do medical students feel training in didactic skills is currently lacking in their medical curriculum?

Generally speaking, interns judged their medical schools’ opportunities for didactic training as insufficient (36.4 %) to adequate (38.1 %). Only 6.3 % of the survey’s respondents said the opportunities at their medical school were good. These were mostly respondents from the medical school of Utrecht. Furthermore, a large proportion, 19.3 %, of medical interns were not aware of the opportunities for training in didactic skills.

##### Do medical students feel a need for education in didactic skills?

Of respondents, 55 % shared the view that their medical school should create additional possibilities for training in didactic skills. Most of them said no such education is currently being offered. Other respondents believed the existing education is of insufficient quality. Some respondents felt that the education on offer should be mandatory.

Only 4.2 % believed it is not necessary to increase the opportunities for training in didactic skills. Of the respondents, 18.7 % said this training is well organized at their medical school and needs no improvement. Little less than a quarter (21.9 %) said it is unclear what training in didactic skills their medical school’s curriculum has on offer.

**Fig. 2:**
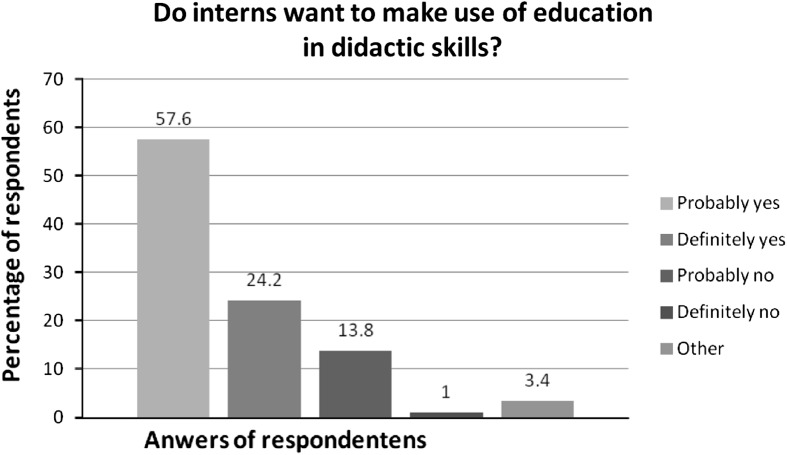
Willingness of students to participate in education in didactic skills

Figure 2 shows the extent to which medical interns would like to participate in training in didactic skills, if they had the opportunity to participate. The majority of students would take part in training in didactic skills if the medical school were to offer them a training programme. The students who definitely or probably want to participate in training in didactic skills together account for 81.8 % of respondents.

### The most suitable teaching method

Being observed while teaching was considered to be the most suitable way to develop didactic skills by 74.1 % of respondents followed by seminars to discuss didactic skills as judged by 66 % of respondents. Lectures about didactics were thought to be less suitable; only 15.2 % of respondents considered this to be an effective method.

## Conclusion and discussion

The current study illustrates that medical interns consider doctors’ didactic skills to be very important to learn from during their internships. Medical interns consider being able to give feedback as the most important skill for doctors in the clinic. Interns believe doctors should not only have the necessary didactic skills but that they are also willing to work on their own teaching skills. The majority of interns report that education in didactic skills should have a higher priority during medical education. Especially teaching while being observed is considered a valuable way of learning didactic skills.

The emphasis in the undergraduate medical curricula of the eight Dutch medical schools varies considerably, though no faculty except Utrecht provides more than ten hours of training in didactic skills [3]. It is striking that some medical schools do not provide any education in didactic skills, since a programme of only four hours ‘teaching to teach’ can make a significant difference in meeting the needs of students [11]. The differences between the medical schools may explain the variation in satisfaction regarding education in didactic skills among the respondents of the survey. The relative overrepresentation of respondents from Utrecht University, the only faculty with a comprehensive elective programme for didactic skills, has positively skewed the data [12]. When interns from Utrecht University were excluded from analysis, the opinion of interns about education in didactics showed a higher degree of dissatisfaction.

Results from previous studies have shown that training in didactic skills in the basic curriculum can successfully teach students those skills. The current study showed the need expressed by medical interns to be more extensively prepared in didactics. The LOCA believes that the timing of this training in the curriculum is of interest too. The LOCA advocates implementing training in didactic skills in the Master’s and not the Bachelor’s phase of the medical degree. According to our research, interns seem to be very willing to receive this education despite the lack in education in didactic skills at the moment. We believe the Master’s phase is the appropriate moment for this education as the moment when these skills will be used as a doctor is closer than for Bachelor students. Therefore those skills will be fresh in their memory when implementing them in medical practice with students. Moreover, Master students are in a later phase of their studies with more medically relevant knowledge and have more ‘educational freedom’ to apply the skills.

## Recommendation

Considering the results of the survey, the LOCA advises that training in didactic skills should be part of the undergraduate curriculum at all medical schools. The LOCA believes that training in didactic skills should be offered in particular in the Master’s phase of the study medicine.

## Essentials

- Medical interns consider didactic skills to be very important.
- Giving appropriate feedback is the most important didactic skill according to medical interns.
- Medical interns are willing to work on their own teaching skills.
- LOCA advises that education in didactic skills should be part of the undergraduate curriculum of all medical schools.
